# An individualized prognostic signature for gastric cancer patients treated with 5-Fluorouracil-based chemotherapy and distinct multi-omics characteristics of prognostic groups

**DOI:** 10.18632/oncotarget.7087

**Published:** 2016-01-30

**Authors:** Xiangyu Li, Hao Cai, Weicheng Zheng, Mengsha Tong, Hongdong Li, Lu Ao, Jing Li, Guini Hong, Mengyao Li, Qingzhou Guan, Sheng Yang, Da Yang, Xu Lin, Zheng Guo

**Affiliations:** ^1^ Department of Bioinformatics, Key Laboratory of Ministry of Education for Gastrointestinal Cancer, The School of Basic Medical Sciences, Fujian Medical University, Fuzhou, China; ^2^ Department of Medical Oncology, Fujian Medical University Union Hospital, Fuzhou, China; ^3^ Department of Pharmaceutical Sciences, University of Pittsburgh, Pittsburgh, USA

**Keywords:** gastric cancer, 5-Fluorouracil-based chemotherapy, gene expression, drug resistance, prognostic signature

## Abstract

5-Fluorouracil (5-FU)-based chemotherapy is currently the first-line treatment for gastric cancer. In this study, using gene expression profiles for a panel of cell lines with drug sensitivity data and two cohorts of patients, we extracted a signature consisting of two gene pairs (*KCNE2* and *API5*, *KCNE2* and *PRPF3*) whose within-sample relative expression orderings (REOs) could robustly predict prognoses of gastric cancer patients treated with 5-FU-based chemotherapy. This REOs-based signature was insensitive to experimental batch effects and could be directly applied to samples measured by different laboratories. Taking this unique advantage of the REOs-based signature, we classified gastric cancer samples of The Cancer Genome Atlas (TCGA) into two prognostic groups with distinct transcriptional characteristics, circumventing the usage of confounded TCGA survival data. We further showed that the two prognostic groups displayed distinct copy number, gene mutation and DNA methylation landscapes using the TCGA multi-omics data. The results provided hints for understanding molecular mechanisms determining prognoses of gastric cancer patients treated with 5-FU-based chemotherapy.

## INTRODUCTION

Gastric cancer is often diagnosed in advanced stage [1], and 5-FU-based chemotherapy is currently recommended as the first-line treatment [2]. As the overall response rate is only about 20-40% [3], it is urgent to develop a signature to recognize patients who cannot benefit from 5-FU-based chemotherapy and recommend them to other chemotherapy regimens. A number of previous studies focused on genes related to 5-FU metabolism (*TS, TP, DPD*), DNA repair (*ERCC1, ERBB2*) or apoptosis (*BCL2, BAX*) to find signatures of 5-FU resistance [4–8] but few have been validated [9]. Then, many studies have turned to use gene expression profiles to identify prognostic signatures for chemo-treated gastric cancer patients, usually based on risk scores summarized from the expression measurements of signature genes [10, 11]. However, this type of signatures cannot be applied directly to independent inter-laboratory data because their applications require pre-setting risk thresholds which are sensitive to experimental batch effects [12]. Although many batch effect correction algorithms and data normalization methods have been proposed, they can hardly correct such biases and even distort the real biological signals [13]. Even if it would be possible to pre-collect a set of samples to measure together with a particular sample for data normalization, the risk-score based signatures would still have a critical limitation that the risk classification of a sample will change with the uncertain risk compositions of the other samples adopted for normalization together, as systematically revealed in our recent work [14]. In contrast, it has been reported that the within-sample relative expression orderings (REOs) of genes are insensitive to experimental batch effects and invariant to monotonic data normalization [15]. With this unique advantage, the REOs-based signatures can perform robustly across datasets produced by different laboratories and allow application at the individual levels [16]. Therefore, it is worthy adopting the REOs-based approach to extract robust prognostic signatures for clinical application.

Notably, researchers often firstly identify prognostic signatures of overall survival (OS) or relapse risk for chemo-treated patients, and then prove the drug benefit predictive value by showing that these signatures could not predict prognoses of patients not receiving chemotherapy [11, 17, 18]. However, this approach is challenged by the argument that patients receiving and not receiving the chemotherapy may have systemic differences in malignant degree of tumor or corporeity [19]. To increase the relevance of prognostic signatures to drug-resistance, Kim et al. [17] pre-selected “drug-resistance” genes from differentially expressed genes (DEGs) between non-responders and responders of patients with the chemotherapy treatment. However, because the tissue samples of non-responders were dissected from patients after the chemotherapy treatment, these DEGs may mainly reflect tissue's response to drug stimuli rather than drug resistance [20]. The same problem exists when pre-selecting DEGs between drug-induced resistant cell lines and parental cell lines [21, 22].

Another major problem in studies for extracting drug resistance signatures for a single drug on clinical trials is that currently combination administration of drugs is conventionally used for cancer chemotherapy [23]. In such a situation, human cancer-derived cell line models provide the only chance to identify drug resistance signature for a single drug [24–26] although the clinical relevance of cancer cell line models remains controversial [27]. Recently, we have proved that if two chemo-regimens shared one or several drugs, then the overlaps between their clinically relevant drug resistance genes (CRGs), defined as the genes differentially expressed in the non-responders compared with responders respectively for the two chemo-regimens, should be (or largely be) the CRGs for the shared single or multiple drugs, given that the drugs used in combination have no (or limited) antagonistic effects [28]. Thus, if we could firstly identify a set of genes positively or negatively associated with 5-FU GI_50_ (50% Growth Inhibition) from gastric cancer cell lines and prove that they are correspondingly negatively or positively associated with prognoses of patients treated with a chemo-regimen including 5-FU as a component, then these genes should be CRGs for 5-FU shared by the cancer cell models and clinical chemo-regimen, given that patients with poor or good prognoses should largely represent non-responders or responders to 5-FU treatment. In this process, the clinical relevance of the cancer cell line models could be evaluated by statistical evidence of concordance analysis (see Materials and Methods) that the genes positively or negatively correlated with 5-FU GI_50_ values of the cell lines were non-randomly negatively or positively correlated with prognoses of patients treated with 5-FU-based chemotherapy.

In this study, by pre-selecting genes correlated with both 5-FU GI_50_ of gastric cancer cells and OS of patients treated with 5-FU-based chemotherapy, we extracted and validated a prognostic signature consisting of two gene pairs. The within-sample REOs of these gene pairs could robustly stratify patients into distinct prognostic groups. Using the robust REOs-based signature, we classified the gastric cancer samples of The Cancer Genome Atlas (TCGA) [29] into two groups. Then, instead of analyzing the TCGA samples' survival data which were confounded with complex chemotherapy regimens and treatment cycles [30], we confirmed that the two identified groups of TCGA samples represented the prognostic groups by evidence that they had the same distinct transcriptional characteristics with the prognostics groups identified in the validation dataset. This strategy enabled us to exploit the TCGA multi-omics data to reveal the distinct copy number, gene mutation and DNA methylation landscapes of the prognostic groups.

## RESULTS

### Extraction of the REOs-based 5-FU-relevant prognostic signature

**Figure 1:**
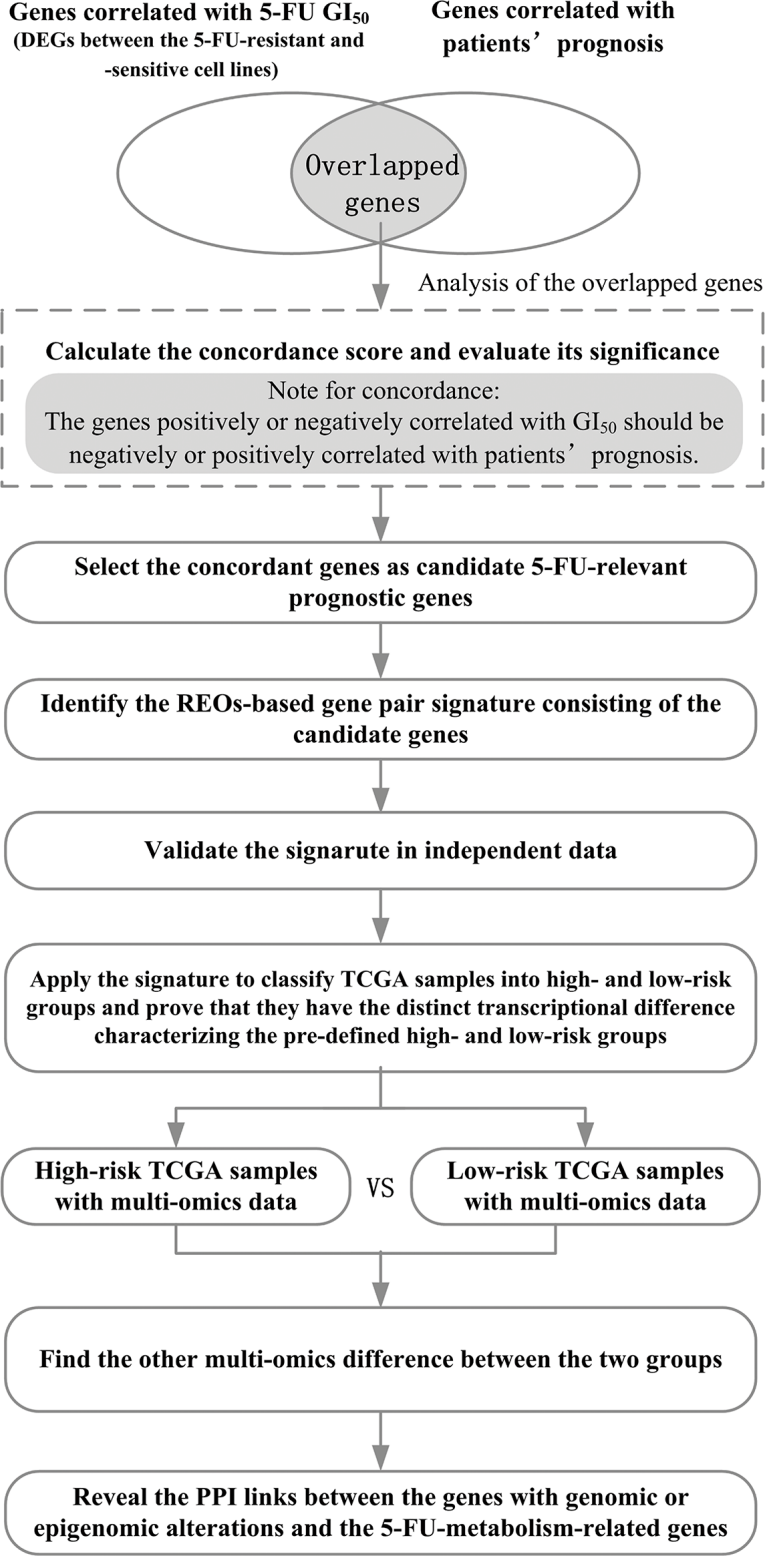
The flowchart for depicting the development, validation and application of the 5-FU-relevant prognostic signature

We hypothesized that genes which are differentially expressed between 5-FU-resistant and -sensitive cell lines while also showing concordant correlation with prognosis in patients could robustly predict prognoses of gastric cancer patients treated with 5-FU-based chemotherapy (Figure 1). Tan et al. [31] classified 28 gastric cancer cell lines into two subtypes which had significantly different average 5-FU GI_50_ values [32], and we defined them as 5-FU-resistant subtype and 5-FU-sensitive subtype, respectively. Based on the gene expression profiles of these cell lines (GSE22183, Table 1), 2,175 DEGs were detected (Student's *t*-test, FDR < 20%) between the two subtypes. Notably, 5 of the 17 cells in the 5-FU-resistant subtype had GI_50_ values below the median of the GI_50_ values of the 28 cells while 2 of the 11 cells in the 5-FU-sensitive subtype had GI_50_ values above the median. When reclassifying these 5 and 2 cell lines into the 5-FU-sensitive and FU-resistant groups, respectively, no DEGs could be detected between the two groups with the same 20% FDR control. Thus, we chose to use the DEGs between the primary subtypes defined by Tan et al. [31] for the 28 cell lines as candidates of 5-FU resistance relevant genes. Statistically, these DEGs should include the genes associated with 5-FU resistance given that most of 5-FU-resistant and 5-FU-sensitive cells were correctly identified. Then, to ensure the relevance of the candidate genes to 5-FU resistance, we further extracted genes correlated with 5-FU GI_50_ values of the cells from these DEGs. In this regard, from the 2,175 DEGs between the two subtypes, we were able to extract 100 genes whose expression levels were significantly correlated with 5-FU GI_50_ values of the 28 cell lines with a reasonable statistical control (FDR < 20%, Pearson correlation analysis). Notably, without the process of preselecting candidates of 5-FU resistance genes, no genes could be found to be significantly correlated with 5-FU GI_50_ values of the 28 cell lines at an acceptable FDR control level (e.g., FDR < 20%) due to the conservativeness of multiple testing correction. Then, based on the gene expression profiles of 35 gastric cancer patients treated with 5-FU-based chemotherapy extracted from the GSE15459 dataset (Table 1) [31], denoted as GC35, we found 14 of the 100 GI_50_-related genes tended to be significantly associated with patients' OS (univariate Cox model, *P* < 0.05). The concordance score of the clinical relevance of these 14 genes was 100%, which was unlikely to be observed by chance (binomial distribution test, *P* < 6.10E–05; see Materials and Methods). In the following analyses, we focused on analyzing nine of the 14 genes, which were also measured in the validation GSE14208 dataset produced by the Affymetrix U133A 2.0 platform (Table 1).

**Table 1:** The datasets of gastric cancer cell lines and tissues analyzed in this study

Accession	Size	Omics	Platform	Stage	Treatment
**Samples of gastric cancer cell lines**					
GSE22183^a^	28	mRNA	AffymetrixU133 Plus 2.0	-	5-FU
**Samples of gastric cancer tissues**					
GSE15459^b^	35	mRNA	AffymetrixU133 Plus 2.0	I–IV	5-FU-based
GSE14208^c^	123	mRNA	AffymetrixU133A 2.0	IV	5-FU plus cisplatin
GSE15459^b^	130	mRNA	AffymetrixU133 Plus 2.0	I–IV	Surgery alone
TCGA^d^	329	mRNA	IlluminaHiSeqRNASeq	I–IV	Mixed
TCGA^e^	327	Copy number	Genome Wide SNP 6.0	I–IV	Mixed
TCGA^e^	289	Somatic mutation	IlluminaGADNASeq/IlluminaHiSeq	I–IV	Mixed
TCGA^e^	293	DNA methylation	HumanMethylation450	I–IV	Mixed

Abbreviation: 5-FU, 5-Fluorouracil;

a The gene expression profiles of 28 gastric cancer cell lines with 5-FU GI_50_ data in this dataset were analyzed. 17 and 11 cell lines were defined as 5-FU-resistant and 5-FU-sensitive, respectively.

b In this dataset, there were 35 samples of patients treated with 5-FU-based chemotherapy and 130 samples of patients treated with surgery alone. These two groups of samples were analyzed. The clinical information of patients was kindly provided by Dr. Ju-Seog Lee.

c The survival data of patients was kindly provided by Dr. Jeffrey E. Green.

d Only 329 TCGA samples of patients in stage I-IV with mRNA-seq profiles, measured by IlluminaHiSeq RNASeq, were analyzed.

e 327, 289 and 293 samples among the 329 TCGA samples with mRNA-seq profiles also had copy number, somatic mutation and DNA methylation data produced by the corresponding platforms, respectively.

**Figure 2:**
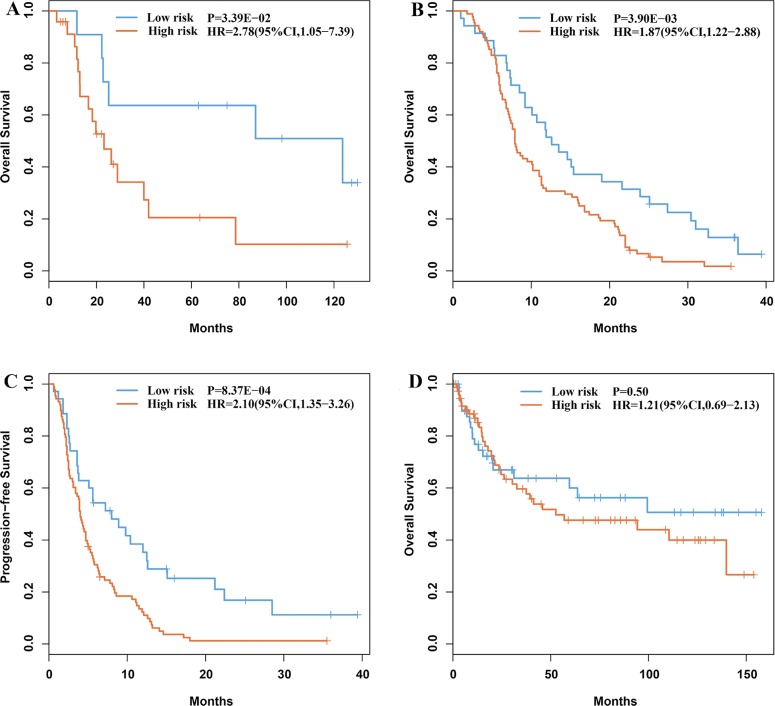
Kaplan-Meier estimates of overall survival and time to progression of the prognostic groups identified by the signature (**A**) Overall survival curves for the GC35 dataset. (**B**) Overall survival curves for the GC123 dataset. (**C**) Time to progression curves for the GC123 dataset. (**D**) Overall survival curves of the 130 gastric cancer patients treated with surgery alone.

For every two of the nine candidate genes, according to their within-sample REO, we classified the 35 gastric cancer samples of the GC35 dataset into two groups and then evaluated whether they had significantly different OS (see Materials and Methods). Using univariate Cox model, we found two gene pairs (*KCNE2* and *PRPF3*, *KCNE2* and *API5*) whose REOs were likely to be associated with patients' OS (*P* < 0.05). *KCNE2* had lower expression level than both *PRPF3* and *API5* in the high-risk group, whereas the REOs were reversed in the low-risk group. Thus, a simple rule was used to classify patients: a patient would be predicted to be of high risk if and only if *KCNE2* had lower expression level than both *PRPF3* and *API5*. According to this rule, 24 and 11 of the 35 samples were classified into the high- and low-risk groups, respectively, and the former had significantly shorter OS than the latter (HR = 2.78, 95%CI 1.05−7.39, log-rank *P* = 3.39E-02, Figure 2A). A multivariate Cox analysis showed that the signature still tended to be prognostic after adjusting for stage, grade and gender even though the size of the GC35 dataset was small (*P* = 0.11, Table 2). As the 35 patients were treated with 5-FU combined with other drugs after surgery, we hypothesized that the high-risk patients could benefit from neither 5-FU nor the other drugs used in combination, whereas the majority of low-risk patients could benefit from 5-FU-based chemotherapy. Thus, we defined these two gene pairs as 5-FU-relevant prognostic signature.

**Table 2:** Univariate and multivariate Cox regression analysis for the 5-FU-relevant prognostic signature

Variables	Univariate model		Multivariate model	
	HR (95%CI)	*P*	HR (95%CI)	*P*
The Prognostic signature				
Low-risk	1 [Reference]		1 [Reference]	
High-risk	2.78 (1.05–7.39)	3.39E–02	2.40 (0.83–6.96)	0.11
Tumor stage				
I–II	1 [Reference]		1 [Reference]	
III–IV	2.05 (0.79–5.29)	0.14	1.40 (0.47–4.11)	0.55
Tumor grade				
Moderate	1 [Reference]		1 [Reference]	
Poor/Undifferentiated	1.95 (0.66–5.81)	0.23	1.79 (0.56–5.72)	0.32
Gender				
Female	1 [Reference]		1 [Reference]	
Male	0.74 (0.29–1.92)	0.55	0.64 (0.21–1.96)	0.43

Abbreviation: HR, hazard ratio; CI, confidence interval.

It has been reported that overexpression of *KCNE2*, which encodes a member of potassium channel on plasma membrane, can facilitate cell apoptosis by mediating K^+^ efflux [33]. Overexpression of *API5*, which encodes an apoptosis inhibitory protein, is related to poor prognosis in various cancers [34]. *PRPF3* encodes a constitutive protein associated with U4 and U6 small nuclear ribonucleoproteins (snRNPs) which make up spliceosome, and abnormal splicing activity is associated with 5-FU efficacy [35].

With the same approach, we have also analyzed the clinical relevance of cisplatin IC_50_ (50% Inhibitory Concentration)-related genes based on data of gastric cancer cell lines but failed to find their correlations with prognosis (see Supplementary Results). This result seems to be consistent with previous reports that no significantly different OS was observed between the 5-FU and cisplatin combination chemotherapy arm and 5-FU alone arm [36, 37]. Therefore, cisplatin therapeutic significance for gastric cancer should be further investigated.

### Validation of the REOs-based 5-FU-relevant prognostic signature

We validated the signature in the GSE14208 dataset (Table 1), denoted as GC123, which included data for 118 gastric cancer patients treated with 5-FU in combination with cisplatin and five patients treated with capecitabine in combination with cisplatin, respectively [17]. As capecitabine is a fluorouracil pro-drug, we collectively regarded the chemo-regimens of the GC123 dataset as 5-FU-based chemotherapy. The signature predicted 88 and 35 of the 123 patients into high- and low-risk groups, respectively. Compared with the low-risk group, the high-risk group had significantly shorter OS (HR = 1.87, 95%CI 1.22-2.88, log-rank *P* = 3.90E–03, Figure 2B) and time to progression (TTP) (HR = 2.10, 95%CI 1.35−3.26, log-rank *P* = 8.37E–04, Figure 2C). The GC123 dataset lacked the necessary clinical data for multivariate Cox analysis. Alternatively, we proved that the transcriptome difference between the prognostic groups for the stage IV samples identified in this dataset was consistent with the corresponding difference for the 24 stage I–III samples involved in the GC35 dataset. Using Student's *t*-test, with FDR < 20%, we extracted 3,927 DEGs between the high- and low-risk groups of stage IV samples from the GC123 dataset. Among these 3,927 DEGs, 456 genes were found to be deregulated between the 15 high-risk samples and 9 low-risk samples identified from the 24 stage I–III samples in the GC35 dataset (Student's *t*-test, *P* < 0.05). The concordance score of the 456 overlapped DEGs was 98.25%, which was unlikely to happen by chance (binomial distribution test, *P* < 1.11E–16; see Materials and Methods). This result provided evidence that the signature was independent of the disease stage.

Functional enrichment analyses (hypergeometric distribution model, FDR < 10%) revealed that the up-regulated genes in the high-risk group compared with the low-risk group identified from the GC123 dataset were significantly enriched in spliceosome, cell cycle, DNA replication, DNA repair (including mismatch repair, nucleotide excision repair, base excision repair and Homologous recombination) and ECM-receptor interaction, whereas the down-regulated genes were significantly enriched in immune, cell adhesion molecules and drug metabolism related pathways (Supplementary Table S1). The pathways enriched with DEGs between the two groups from the GC35 dataset were all reproducible in GC123 (Supplementary Table S2).

Finally, we applied the signature to predict the survival of the 130 samples of gastric cancer patients treated with surgery alone, which were extracted from the GSE15459 dataset and found that it could not stratify the patients into two groups with significantly different OS (HR = 1.21, 95%CI 0.69−2.13, log-rank *P* = 0.50, Figure 2D). This reflected that the signature was not just prognostic for gastric cancer patients in general but predictive for patient's benefit from 5-FU-based chemotherapy.

### Distinct genomic characteristics of prognostic groups

Applying the prognostic signature to the gene expression profiles of 329 gastric cancer samples documented in TCGA (Table 1), we recognized 286 high-risk patients and 43 low-risk patients. Between the two prognostic groups for patients in stage I, II, III and IV, respectively, we detected 1,323, 3,103, 2,823 and 761 DEGs (Rank Products, FDR < 20%). The four lists of DEGs shared 269, 706, 736 and 182 genes with the 3,927 DEGs from the GC123 dataset for stage IV patients, and the concordance scores were 90.17%, 83.85%, 95.11% and 89.01% (binomial distribution test, all values of *P* < 1.11E-16), respectively. This result provided further evidence that the signature was independent of the disease stage. Among the 329 TCGA tumors, 327 (284 high-risk and 43 low-risk samples) had copy number alteration data; 289 (249 high-risk and 40 low-risk samples) had somatic mutation data; and 293 (252 high-risk and 41 low-risk samples) had DNA methylation data (as described in Table 1). This allowed us to further characterize the two prognostic groups in genome and epigenome.

**Figure 3:**
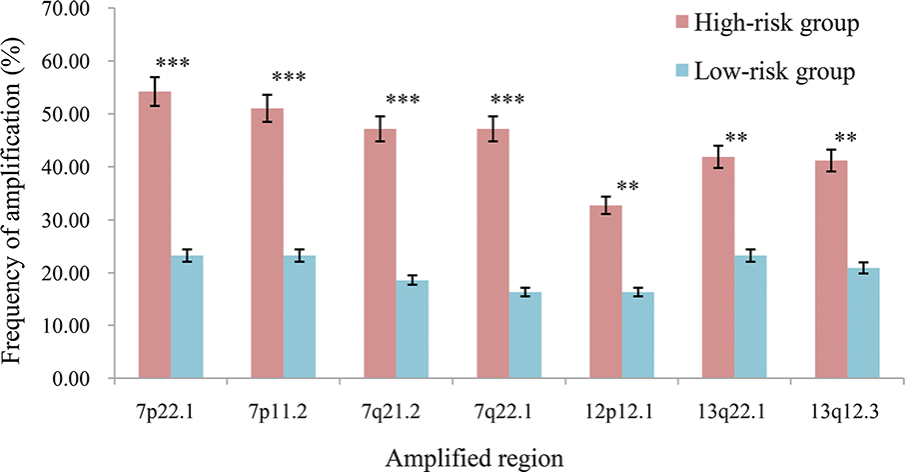
The frequencies of the seven amplified regions in the two prognostic groups ***P < 0.001, **P < 0.05.

**Figure 4:**
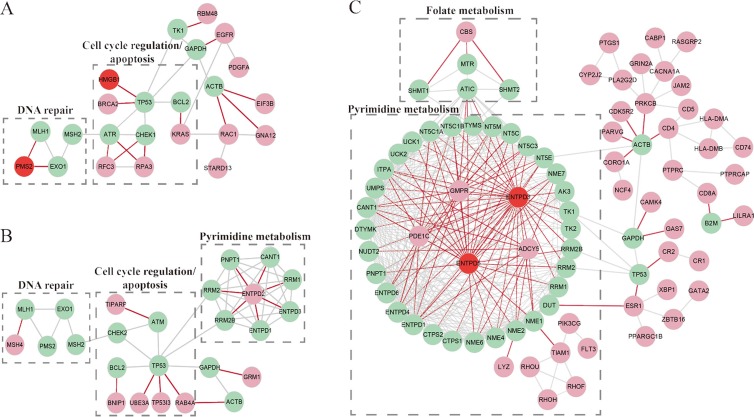
The PPI links between the 5-FU-metabolism-related genes and the genes with genomic or epigenomic alterations characterizing each of the prognostic groups (**A**) The sub-network for the genes frequently amplified in the high-risk group. (**B**) The sub-network for the genes frequently mutated in the low-risk group. (**C**) The sub-network for the hypermethylation-mediated down-regulated genes in the high-risk group. The aquamarine nodes denote the 5-FU-metabolism-ralted genes. The pink nodes denote amplified genes (A), mutation genes (B), or hypermethylation-mediated down-regulation genes (C). The red nodes denote the 5-FU-metabolism-related genes with genomic or epigenomic alterations. The red edges denote the direct PPI links between the aquamarine nodes and the pink or red nodes.

Interestingly, we observed that the high-risk patients had a distinct copy number amplification landscape comparing to the low-risk patients, with significantly higher frequencies (Fisher's exact test, FDR < 20%) of copy number gain at 7p22.1, 7p11.2, 7q21.2, 7q22.1, 13q22.1, 13q12.3 and 12p12.1 (Figure 3), whereas the two prognostic groups had no difference in copy number loss regions. In further integrated analysis with the gene expression data, we found that 85 genes located in the seven amplified regions displayed significant overexpression in the high-risk samples (Spearman rank correlation, FDR < 20%). Functional enrichment analysis (FDR < 10%) showed that these 85 genes were significantly enriched in the “mismatch repair pathway”, indicating that the potentially enhanced ability of mismatch repair could lead to failure of 5-FU-induced DNA damage in the high-risk patients [38]. In the PPI network (see Materials and Methods), 12.94% (11) of the 85 genes with frequent amplification in the high-risk patients had direct PPI links with at least one of the 92 5-FU metabolism-related genes collected by Tan et al. [39], which was significantly higher than the corresponding frequency of 4.05% for the rest 518 genes located in all the amplified regions in the gastric cancer tissues (Fisher's exact test, *P* = 2.41E–03). As shown in Figure 4A, the 11 genes frequently amplified in high-risk patients directly interacted with nine 5-FU metabolism-related genes including *ATR* and *CHEK1* [40] involved in cell cycle regulation, *MLH1*, *PMS2* and *EXO1* [38] involved in DNA repair, and *BCL2* [41] involved in apoptosis (Supplementary Table S3). This result suggested that the high-risk patients had higher malignant degree of tumors.

Comparison of somatic mutation profiles of high- and low-risk samples characterized 156 genes whose mutation frequencies tended to be different between the two prognostic groups (Fisher's exact test, *P* < 0.05). Among them, *LRP1B* was mutated in 31.33% of samples in the high-risk group while its mutation frequency was 15% in the low-risk group. It has been reported that chromosomal, epigenetic and microRNA-mediated inactivation of *LRP1B* increases the growth and invasive capacity of tumor cells [42]. In addition to *LRP1B*, all other 155 genes had higher mutation frequencies in the low-risk group compared with the high-risk group, significantly more than what expected by chance (*P* < 1.11E–16). Functional enrichment analysis (*P* < 0.05) showed that these 155 mutation genes tended to be enriched in Wnt signaling, Ras signaling and Regulation of autophagy pathways, which implied that mutation-induced disturbances of these pathways might promote 5-FU efficacy for the low-risk patients [43–45]. In the PPI network, 5.13% (eight) of the 155 genes frequently mutated in the low-risk group had direct PPI links with at least one of the 92 5-FU metabolism-related genes, which was significantly higher than the corresponding frequency of 0.93% for the rest 18,759 mutated genes without significantly different mutation frequencies between the two prognostic groups (Fisher's exact test, *P* = 1.36E–04). As shown in Figure 4B, the eight mutation genes directly interacted with 13 5-FU metabolism-related genes including *RRM1* involved in pyrimidine metabolism [46], *ATM* [40], *MHL1* and *BCL2* involved in DNA repair, cell cycle regulation and apoptosis (Supplementary Table S4). Therefore, it is possible that these mutation-induced disturbances could facilitate 5-FU efficacy for the low-risk patients.

### Distinct epigenomic characteristics of prognostic groups

Using the high- and low-risk TCGA samples with DNA methylation profiles, we identified 1,480 hypermethylated genes and 1,235 hypomethylated genes in the high-risk group compared with the low-risk group (Rank Products, FDR < 20%), respectively. Among the 1,480 hypermethylated genes, 400 genes were also identified as DEGs between the high-risk group and the low-risk group, and the concordance score of hypermethylation with down-regulation was 81.75%, which was highly unlikely to occur by chance (binomial distribution test, *P* < 1.11E–16; see Materials and Methods). This result suggested that the down-regulation of the concordant genes could be mediated by DNA hypermethylation. These genes were significantly enriched in the “cell adhesion molecules pathway” (FDR < 10%), indicating that hypermethylation-induced suppression of this pathway might contribute to 5-FU-based chemotherapy resistance. Among the 327 hypermethylation-mediated down-regulated genes in the high-risk group, 5.81% (19) had direct PPI links with at least one of the 92 5-FU metabolism-related genes, which was significantly higher than the corresponding frequency of 1.53% for the rest 12,476 genes without concordant hypermethylation with down-regulation in the high-risk group (Fisher's exact test, *P* = 1.83E–06). As shown in Figure 4C, the 19 hypermethylation-mediated down-regulated genes directly interacted with 40 5-FU metabolism-related genes including *NME1* [47] and *TK1*/*2* [48] involved in pyrimidine metabolism, *MTR* and *SHMT1*/*2* [49] involved in folate metabolism (Supplementary Table S5). Folate, as a co-factor, can assist the 5-FU active metabolite flurodeoxyuridine monophosphate (FdUMP) to inhibit thymidylate synthase (TS) from DNA synthesis and repair [48]. The results implied that the hypermethylation-mediated down-regulation of genes in the high-risk patients might disturb the conversion of 5-FU to active metabolites and inhibit 5-FU-induced DNA or RNA damage.

On the other hand, the concordance score of hypomethylation with up-regulation was only 39.63%, providing no evidence of hypomethylation- mediated up-regulation of genes.

## DISCUSSION

In this study, we developed a signature consisting of two gene pairs whose within-sample REOs were prognostic for gastric cancer patients treated with 5-FU-based chemotherapy. This REOs-based signature could perform robustly in independent datasets produced by different laboratories and could be easily applied at the individual levels. With this unique advantage, we were able to transform the transcriptional signature to other omics signature using the TCGA multi-omics data as a pivot. This strategy makes it feasible to explore the genomic and epigenomic characteristics of prognostic groups using the TCGA multi-omics data which would otherwise be largely unsuitable for prognostic analyses because the diverse chemo-regimens could confound the survival outcomes. Our analyses showed that the high-risk patients had frequent amplification of genes affecting DNA repair, cell cycle regulation and apoptosis, indicating that they had high malignant degree of tumors. Meanwhile, the hypermethylation-mediated down-regulation of genes in the high-risk group mainly affected pyrimidine and folate metabolism, which might decrease the conversion of 5-FU to active metabolites and inhibit 5-FU-induced DNA or RNA damage. In contrast, the low-risk patients were characterized with frequent mutation of genes enriched in Wnt signaling, Ras signaling and Regulation of autophagy pathways, implying that these mutation-induced disturbances might facilitate 5-FU efficacy.

We need to clarify that we should not simply conclude that the high-risk patients identified by the prognostic signature are all resistant to 5-FU-based chemotherapy. Some of the high-risk patients' tumor could be indeed resistant to 5-FU-induced DNA or RNA damage [48], while some others might be sensitive to 5-FU but their tumor cell growth ability outperforms the drug efficacy [50]. Despite this problem, such a prognostic signature can still provide valuable information for clinical recommendation of adjuvant chemotherapy. Because all the high-risk patients should have high malignant degree of tumors and the routine clinical chemotherapy might be unable to improve their clinical outcomes, other therapy regimens or a larger dosage of chemotherapy could be recommended to these patients [51]. In contrast, the low-risk patients identified by the signature should include both patients with high malignant degree of tumors who, however, can benefit from chemotherapy and patients with low malignant degree of tumors who could be at low risk without the help of chemotherapy. We could recommend 5-FU-based chemotherapy to these patients. Especially, for the advanced patients with distant metastasis, because the improved survival of the low-risk patients must be attributed to 5-FU-based chemotherapy, the signature could identify the patients who can benefit from 5-FU-based chemotherapy. To recognize the patients who are sensitive or resistant to 5-FU, we need gene expression profiles of patients with explicit information of response, which, however, are currently scarce in public databases. Also, future work is needed to study whether it is possible to design novel drugs targeting the genomic or epigenomic lesions characterizing the patients who cannot benefit from 5-FU-based chemotherapy.

In summary, the discovery that the REOs-based signature could robustly predict prognoses of 5-FU-treated gastric cancer patients will provide a translational biomarker in further stratifying the gastric cancer patients for 5-FU response. The multi-omics characteristics of the high-risk patients would expand our understanding of the mechanisms underlying 5-FU resistance in gastric cancer and provide novel therapeutic targets to overcome 5-FU resistance of gastric patients in the future.

## MATERIALS AND METHODS

### Data and pre-processing

Data for gastric cancer cell lines and tissues were downloaded from the Gene Expression Omnibus (GEO, http://www.ncbi.nlm.nih.gov/geo/) and TCGA (http://cancergenome.nih.gov/), as described in details in Table 1. The raw data (.CEL files) of microarray platforms were processed using the Robust Multichip Average algorithm [52]. Probe IDs were mapped to gene IDs using the corresponding platform files. If multiple probes were mapped to the same gene, the expression value for the gene was summarized as the arithmetic mean of the values of the multiple probes. The mRNA-seq profiles of level 3 for TCGA samples were downloaded from TCGA portal. We removed genes whose expression measurements were at or below a noise threshold of 0.2 reads per kilobase per million mapped reads (RPKM) in at least 75% of samples [29].

Copy number data of level 4 for TCGA samples analyzed by the GISTIC 2.0 algorithm [53] were downloaded from Firehose (https://confluence.broadinstitute.org/display/GDAC/Download). Using the significant regions of gain or loss identified by GISTIC 2.0, we assigned a discrete copy number alteration status to each gene in each sample. Gene mutation data of level 2 and DNA methylation data of level 3 for TCGA samples were downloaded from TCGA portal. For gene mutation data, only the non-synonymous mutations were included in our analysis. By integrating mutation data produced by different platforms, we generated a discrete mutation profile including 18,916 genes. DNA methylation profiles of level 3 provided beta-value for each CpG site in each sample. We focused on the 25,978 CpG sites located at the promoter regions of genes, which were measured by Illumina Infinium Human DNA methylation 450 and 27 platforms. Probes that had any “NA”-masked data points and that were designed for sequences on X and Y chromosomes were removed [29]. Probe IDs were mapped to gene IDs using the corresponding platform file. If multiple probes were mapped to the same gene, the beta-value for this gene was summarized as the arithmetic mean of the values of the multiple probes. Totally, 21,993 CpG sites mapped to 12,803 genes were analyzed in this study.

The human protein-protein interaction (PPI) data including 142,583 distinct interactions and 13,693 human proteins were collected as previously described [54]. The types of interaction relationships between proteins included physical interaction, transcriptional regulation and sequential catalysis.

### Correlation and survival analysis

The Pearson correlation analysis was used to evaluate the correlation of genes expression levels with GI_50_ values of cell lines. The univariate Cox regression model was used to evaluate the correlation of gene expression levels and REOs of gene pairs with OS, and the multivariate Cox regression model was used to evaluate the independent prognostic value of the signature after adjusting for clinical factors including stage, grade and gender. Survival curves were estimated by the Kaplan-Meier method and compared with log-rank test.

### Development of the prognostic signature

Let *Ea* and *Eb* represent the expression levels of two candidate genes, a and b, respectively, we classified cancer samples into two groups according to the within-sample REO (*Ea* > *Eb* or *Ea* < *Eb*) of this gene pair. If the two groups of samples had significantly different OS, then we defined this gene pair as a prognosis-associated gene pair. If the *Ea* > *Eb* REO was associated with poor OS, then this REO voted for high risk; otherwise, low risk. All the prognosis-associated gene pairs were selected as prognostic signature. Finally, a sample was predicted to be of high risk if the REOs of all gene pairs of the signature in this sample voted for high risk; otherwise, low risk.

### Concordance scores

If two lists of DEGs detected separately from two datasets had *k* overlapped genes, among which *s* genes showed the same deregulation directions (up- or down-regulation) in the two DEGs lists, then the concordance score was calculated as *s*/*k*. This score was used to evaluate the consistence of DEGs extracted from independent datasets.

If *k* genes were found to be correlated with both 5-FU GI_50_ of cell lines and patients' OS, among which *s* genes had the same signs positively (or negatively) correlated with 5-FU GI_50_ of cell lines and correspondingly negatively (or positively) correlated with patients' OS, then the concordance score was calculated as *s*/*k*. This score was used to evaluate the clinical relevance of the GI_50_-related genes.

If *k* genes had both methylation and expression changes, among which *s* genes were hypermethylated (or hypomethylated) and correspondingly down-regulated (or up-regulated), then the concordance score was calculated as *s*/*k*. This score was used to evaluate the concordance of hypermethylation (or hypomethylation) with down-regulation (or up-regulation).

The probability of observing a concordance score of *s*/*k* by chance was evaluated by the cumulative binomial distribution model as following:

$$P=1-\sum_{i=0}^{s-1}\left(\begin{array}{c} k\\ i \end{array}\right){\left(P_{e}\right)}^{i}{\left(1-P_{e}\right)}^{k-1}$$

where *P_e_* is the probability of one gene having the concordant relationship between the two lists of genes by chance (here, *P_e_* = 0.5).

### Analysis of genomic and epigenomic data

The Student's *t*-test was used to select DEGs between two groups of samples. The Rank Products algorithm [55], which is insensitive to batch effects, was used to select DEGs and differential DNA methylation genes (DMGs) between two groups of TCGA samples derived from multiple experimental batches.

Fisher's exact test was conducted to extract genes which had significantly different frequencies of copy number alterations and mutation between two groups of TCGA samples. Spearman rank correlation analysis was used to evaluate the correlation between copy number alterations and expression changes after removing the batch effects by ComBat for TCGA samples [56].

### Functional enrichment analysis

Functional enrichment analyses were performed based on KEGG (the Kyoto Encyclopedia of Genes and Genomes) [57]. The hypergeometric distribution model was used to calculate the significance of biological pathways enriched with genes of interest [58]. The Benjamini-Hochberg method was adopted to estimate the false discovery rate (FDR). All statistical analyses were performed using the R software package version 3.0.1.

### The flowchart of the analysis procedure

Figure 1 describes the development, validation and application of the 5-FU-relevant prognostic signature.

## SUPPLEMENTARY MATERIALS TABLES



## Abbreviations

5-Fluorouracil: 5-FU
REOs: Relative Expression Orderings
OS: Overall survival
FDR: False Discovery Rate
HR: Hazard Ratios
CI: Confidence Intervals
PPI: Protein-Protein Interaction
