# Supplementary material for: An individualized prognostic signature for gastric cancer patients treated with 5-Fluorouracil-based chemotherapy and distinct multi-omics characteristics of prognostic groups
Source: Oncotarget. 2016 Jan 30;7(8):8743–55. doi: 10.18632/oncotarget.7087 (PMC4891001; doi:10.18632/oncotarget.7087)
Supplement: Supplementary file 1 [file oncotarget-07-8743-s001.pdf]

An individualized prognostic signature for gastric cancer patients treated with 5-Fluorouracil-based chemotherapy and distinct multi-omics characteristics of prognostic groups

Supplementary Materials

SUPPLEMENTARY RESULTS

Cisplatin therapeutic significance for gastric cancer

The E-MTAB-783 from ArrayExpress (http://www.ebi.ac.uk/arrayexpress/) included 11 gastric cancer cell lines with corresponding cisplatin IC_{50} values. Using Pearson correlation analysis, with FDR < 0.2, no genes were found to be significantly correlated with cispatin IC_{50} values of the 11 gastric cancer cell lines. Therefore, with $P < 0.05$, we identified 555 IC_{50}-related genes. Then, based on the dataset GC123, we found that 42 of the 555 IC_{50}-related genes were tended to be associated with patients' OS (univariate Cox model, $P < 0.05$). However, the concordance score of the clinical relevance of these 42 cisplatin IC_{50}-related genes was only 28.57% (binomial distribution test, $P \approx 1$), implying that cisplatin IC_{50}-related genes might lack correlation with clinical prognoses of patients receiving cisplatin-based chemotherapy. Therefore, cisplatin therapeutic significance for gastric cancer should be further investigated.

Supplementary Table S1: The pathways enriched with DEGs between the two prognostic groups of GC123

Systemic name	Pathway name	Number of annotated DEGs	*P*
The pathways enriched with up-regulated genes			
hsa03013	RNA transport	71	1.11E–16
hsa03040	Spliceosome	59	1.11E–16
hsa04110	Cell cycle	62	1.11E–16
hsa03010	Ribosome	54	3.75E–13
hsa03008	Ribosome biogenesis in eukaryotes	36	5.82E–13
hsa03050	Proteasome	25	1.71E–09
hsa03030	DNA replication	20	1.00E–07
hsa00240	Pyrimidine metabolism	31	1.34E–05
hsa03430	Mismatch repair	13	1.76E–05
hsa03020	RNA polymerase	13	1.08E–04
hsa03420	Nucleotide excision repair	17	3.40E–04
hsa03410	Base excision repair	14	3.96E–04
hsa03015	mRNA surveillance pathway	25	6.87E–04
hsa03022	Basal transcription factors	15	1.00E–03
hsa03440	Homologous recombination	11	4.48E–03

hsa04914	Progesterone-mediated oocyte maturation	23	4.59E–03
hsa00230	Purine metabolism	37	5.31E–03
hsa04512	ECM-receptor interaction	24	7.33E–03
The pathways enriched with down-regulated genes			
hsa04640	Hematopoietic cell lineage	31	4.69E–07
hsa04672	Intestinal immune network for IgA production	19	1.00E–06
hsa00830	Retinol metabolism	18	3.29E–06
hsa04514	Cell adhesion molecules (CAMs)	37	3.88E–06
hsa04650	Natural killer cell mediated cytotoxicity	34	2.61E–05
hsa00982	Drug metabolism - cytochrome P450	18	6.74E–05
hsa00980	Metabolism of xenobiotics by cytochrome P450	18	2.22E–04
hsa00601	Glycosphingolipid biosynthesis - lacto and neolacto series	11	2.31E–04
hsa00340	Histidine metabolism	10	4.85E–04
hsa04612	Antigen processing and presentation	21	6.45E–04
hsa00650	Butanoate metabolism	10	7.48E–04
hsa04666	Fc gamma R-mediated phagocytosis	23	1.23E–03
hsa00071	Fatty acid degradation	14	1.28E–03
hsa00380	Tryptophan metabolism	12	1.52E–03
hsa04146	Peroxisome	20	2.03E–03
hsa04971	Gastric acid secretion	18	4.88E–03
hsa00561	Glycerolipid metabolism	13	5.02E–03
hsa04660	T cell receptor signaling pathway	24	6.61E–03
hsa04662	B cell receptor signaling pathway	18	6.84E–03
hsa00051	Fructose and mannose metabolism	10	7.55E–03
hsa00040	Pentose and glucuronateinterconversions	7	9.81E–03
hsa00512	Mucin type O-Glycan biosynthesis	7	9.81E–03

Abbreviation: DEGs, differential expressed genes.

Supplementary Table S2: The pathways enriched with DEGs between the two prognostic groups of GC35

Systemic name	Pathway name	Number of annotated DEGs	*P*
The pathways enriched with up-regulated genes			
hsa04672	Intestinal immune network for IgA production	5	8.26E–04
hsa04640	Hematopoietic cell lineage	7	9.15E–04
hsa04971	Gastric acid secretion	6	1.21E–03
hsa00982	Drug metabolism - cytochrome P450	5	1.71E–03
hsa00051	Fructose and mannose metabolism	4	2.01E–03

Abbreviation: DEGs, differential expressed genes.

Supplementary Table 3: The 11 amplified genes with direct PPI interaction with 5-FU metabolism-related genes

Amplified genes	Gene ID	Region	5-FU-metabolism-related genes	Gene ID	5-FU-metabolism-related pathways
PMS2[a]	5395	-	*PMS2*	5395	DNArepair/apoptosis/cell cycle regulation
HMGB1[a]	3146	-	*HMGB1*	3146	DNArepair/apoptosis/cell cycle regulation
BRCA2	675	13q12.3	*TP53*	7157	DNArepair/apoptosis/cell cycle regulation
HMGB1[a]	3146	13q12.3	*TP53*	7157	DNArepair/apoptosis/cell cycle regulation
KRAS	3845	12p12.1	*BCL2*	596	DNArepair/apoptosis/cell cycle regulation
PMS2[a]	5395	7p22.1	*MLH1*	4292	DNArepair/apoptosis/cell cycle regulation
PMS2[a]	5395	7p22.1	*EXO1*	9156	DNArepair/apoptosis/cell cycle regulation
RFC3	5983	13q12.3	*ATR*	545	DNArepair/apoptosis/cell cycle regulation
RFC3	5983	13q12.3	*CHEK1*	1111	DNArepair/apoptosis/cell cycle regulation
RPA3	6119	7p22.1	*ATR*	545	DNArepair/apoptosis/cell cycle regulation
RPA3	6119	7p22.1	*CHEK1*	1111	DNArepair/apoptosis/cell cycle regulation
GNA12	2768	7p22.1	*ACTB*	60	Housekeeping (cytoskeleton)
RAC1	5879	7p22.1	*ACTB*	60	Housekeeping (cytoskeleton)
EIF3B	8662	7p22.1	*ACTB*	60	Housekeeping (cytoskeleton)
EGFR	1956	7p11.2	*GAPDH*	2597	Housekeeping (glycolysis)
RBM48	84060	7q21.2	*TK1*	7083	Pyrimidine metabolism

[a]This gene is 5-FU metabolism-related.

Supplementary Table S4: The 8 mutation genes with direct PPI interaction with 5-FU metabolism-related genes

Mutation genes	Gene ID	5-FU-metabolism-related genes	Gene ID	5-FU-metabolism-related pathways
ENTPD2	954	*ENTPD1*	953	Pyrimidine metabolism
ENTPD2	954	*ENTP*D3	956	Pyrimidine metabolism
ENTPD2	954	*RRM1*	6240	Pyrimidine metabolism
ENTPD2	954	*RRM2*	6241	Pyrimidine metabolism
ENTPD2	954	*RRM2B*	50484	Pyrimidine metabolism
ENTPD2	954	*PNPT1*	87178	Pyrimidine metabolism
ENTPD2	954	*CANT1*	124583	Pyrimidine metabolism
GRM1	2911	*GAPDH*	2597	Housekeeping (glycolysis)
RAB4A	5867	*ACTB*	60	Housekeeping (cytoskeleton)
BNIP1	662	*BCL2*	596	DNArepair/apoptosis/cell cycle regulation
MSH4	4438	*MLH1*	4292	DNArepair/apoptosis/cell cycle regulation
RAB4A	5867	*TP53*	7157	DNA repair/apoptosis/cell cycle regulation
UBE3A	7337	*TP53*	7157	DNA repair/apoptosis/cell cycle regulation
TP53I3	9540	*TP53*	7157	DNA repair/apoptosis/cell cycle regulation
TIPARP	25976	*ATM*	472	DNA repair/apoptosis/cell cycle regulation

Supplementary Table S5: The 19 hypermethylation-mediated down-regulation genes with direct PPI interaction with 5-FU metabolism-related genes

Hypermethylation-mediated down-regulation genes	Gene ID	5-FU-metabolism-related genes	Gene ID	5-FU-metabolism-related pathways
ENTPD3[a]	956	*TYMS*	7298	Transporters
ENTPD3[a]	956	*ENTPD3*	956	Pyrimidine metabolism
ENTPD5[a]	957	*ENTPD5*	957	Pyrimidine metabolism
ADCY5	111	*NUDT2*	318	Pyrimidine metabolism
ADCY5	111	*ENTPD1*	953	Pyrimidine metabolism
ADCY5	111	*ITPA*	3704	Pyrimidine metabolism
ADCY5	111	*NME1*	4830	Pyrimidine metabolism
ADCY5	111	*NME2*	4831	Pyrimidine metabolism
ADCY5	111	*NME4*	4833	Pyrimidine metabolism
ADCY5	111	*NME6*	10201	Pyrimidine metabolism
ADCY5	111	*NME7*	29922	Pyrimidine metabolism
ADCY5	111	*CANT1*	124583	Pyrimidine metabolism

ENTPD3[a]	956	*NUDT2*	318	Pyrimidine metabolism
ENTPD3[a]	956	*ENTPD1*	953	Pyrimidine metabolism
ENTPD3[a]	956	*ENTPD6*	955	Pyrimidine metabolism
ENTPD [a]	956	*CTPS1*	1503	Pyrimidine metabolism
ENTPD3[a]	956	*DTYMK*	1841	Pyrimidine metabolism
ENTPD3[a]	956	*ITPA*	3704	Pyrimidine metabolism
ENTPD3[a]	956	*NME1*	4830	Pyrimidine metabolism
ENTPD3[a]	956	*NME2*	4831	Pyrimidine metabolism
ENTPD3[a]	956	*NME4*	4833	Pyrimidine metabolism
ENTPD3[a]	956	*NT5E*	4907	Pyrimidine metabolism
ENTPD3[a]	956	*RRM1*	6240	Pyrimidine metabolism
ENTPD3[a]	956	*RRM2*	6241	Pyrimidine metabolism
ENTPD3[a]	956	*TK1*	7083	Pyrimidine metabolism
ENTPD3[a]	956	*TK2*	7084	Pyrimidine metabolism
ENTPD3[a]	956	*UCK2*	7371	Pyrimidine metabolism
ENTPD3[a]	956	*UMPS*	7372	Pyrimidine metabolism
ENTPD3[a]	956	*ENTPD4*	9583	Pyrimidine metabolism
ENTPD3[a]	956	*NME6*	10201	Pyrimidine metabolism
ENTPD3[a]	956	*NME7*	29922	Pyrimidine metabolism
ENTPD3[a]	956	*NT5C*	30833	Pyrimidine metabolism
ENTPD3[a]	956	*RRM2B*	50484	Pyrimidine metabolism
ENTPD3[a]	956	*AK3*	50808	Pyrimidine metabolism
ENTPD3[a]	956	*NT5C3*	51251	Pyrimidine metabolism
ENTPD3[a]	956	*CTPS2*	56474	Pyrimidine metabolism
ENTPD3[a]	956	*NT5M*	56953	Pyrimidine metabolism
ENTPD3[a]	956	*UCK1*	83549	Pyrimidine metabolism
ENTPD3[a]	956	*NT5C1A*	84618	Pyrimidine metabolism
ENTPD3[a]	956	*PNPT1*	87178	Pyrimidine metabolism
ENTPD3[a]	956	*NT5C1B*	93034	Pyrimidine metabolism
ENTPD3[a]	956	*CANT1*	124583	Pyrimidine metabolism
ENTPD5[a]	957	*ENTPD1*	953	Pyrimidine metabolism
ENTPD5[a]	957	*ITPA*	3704	Pyrimidine metabolism
ENTPD5[a]	957	*NME1*	4830	Pyrimidine metabolism
ENTPD5[a]	957	*NME2*	4831	Pyrimidine metabolism
ENTPD5[a]	957	*NME4*	4833	Pyrimidine metabolism
ENTPD5[a]	957	*NT5E*	4907	Pyrimidine metabolism

ENTPD5[a]	957	*RRM1*	6240	Pyrimidine metabolism
ENTPD5[a]	957	*RRM2*	6241	Pyrimidine metabolism
ENTPD5[a]	957	*UCK2*	7371	Pyrimidine metabolism
ENTPD5[a]	957	*UMPS*	7372	Pyrimidine metabolism
ENTPD5[a]	957	*NME6*	10201	Pyrimidine metabolism
ENTPD5[a]	957	*NME7*	29922	Pyrimidine metabolism
ENTPD5[a]	957	*NT5C*	30833	Pyrimidine metabolism
ENTPD5[a]	957	*RRM2B*	50484	Pyrimidine metabolism
ENTPD5[a]	957	*AK3*	50808	Pyrimidine metabolism
ENTPD5[a]	957	*NT5C3*	51251	Pyrimidine metabolism
ENTPD5[a]	957	*NT5M*	56953	Pyrimidine metabolism
ENTPD5[a]	957	*UCK1*	83549	Pyrimidine metabolism
ENTPD5[a]	957	*NT5C1A*	84618	Pyrimidine metabolism
ENTPD5[a]	957	*PNPT1*	87178	Pyrimidine metabolism
ENTPD5[a]	957	*NT5C1B*	93034	Pyrimidine metabolism
ENTPD5[a]	957	*CANT1*	124583	Pyrimidine metabolism
ESR1	2099	*DUT*	1854	Pyrimidine metabolism
GMPR	2766	*ENTPD1*	953	Pyrimidine metabolism
GMPR	2766	*ENTPD6*	955	Pyrimidine metabolism
GMPR	2766	*ITPA*	3704	Pyrimidine metabolism
GMPR	2766	*NT5E*	4907	Pyrimidine metabolism
GMPR	2766	*ENTPD4*	9583	Pyrimidine metabolism
GMPR	2766	*NT5C*	30833	Pyrimidine metabolism
GMPR	2766	*NT5C3*	51251	Pyrimidine metabolism
GMPR	2766	*NT5M*	56953	Pyrimidine metabolism
GMPR	2766	*NT5C1A*	84618	Pyrimidine metabolism
GMPR	2766	*NT5C1B*	93034	Pyrimidine metabolism
GMPR	2766	*CANT1*	124583	Pyrimidine metabolism
LYZ	4069	*NME2*	4831	Pyrimidine metabolism
PDE1C	5137	*ENTPD1*	953	Pyrimidine metabolism
PDE1C	5137	*ENTPD6*	955	Pyrimidine metabolism
PDE1C	5137	*ITPA*	3704	Pyrimidine metabolism
PDE1C	5137	*NT5E*	4907	Pyrimidine metabolism
PDE1C	5137	*ENTPD4*	9583	Pyrimidine metabolism
PDE1C	5137	*NT5C*	30833	Pyrimidine metabolism
PDE1C	5137	*NT5C3*	51251	Pyrimidine metabolism

PDE1C	5137	*NT5M*	56953	Pyrimidine metabolism
PDE1C	5137	*NT5C1A*	84618	Pyrimidine metabolism
PDE1C	5137	*NT5C1B*	93034	Pyrimidine metabolism
PDE1C	5137	*CANT1*	124583	Pyrimidine metabolism
TIAM1	7074	*NME1*	4830	Pyrimidine metabolism
CBS	875	*MTR*	4548	Folate metabolism
CBS	875	*SHMT1*	6470	Folate metabolism
CBS	875	*SHMT2*	6472	Folate metabolism
ENTPD[a]	956	*ATIC*	471	Folate metabolism
ENTPD5[a]	957	*ATIC*	471	Folate metabolism
GMPR	2766	*ATIC*	471	Folate metabolism
CD8A	925	*B2M*	567	Housekeeping (MHC I class molecule)
LILRA1	11024	*B2M*	567	Housekeeping (MHC I class molecule)
CAMK4	814	*GAPDH*	2597	Housekeeping (glycolysis)
GAS7	8522	*GAPDH*	2597	Housekeeping (glycolysis)
CD4	920	*ACTB*	60	Housekeeping (cytoskeleton)
PRKCB	5579	*ACTB*	60	Housekeeping (cytoskeleton)
CDK5R2	8941	*ACTB*	60	Housekeeping (cytoskeleton)
CORO1A	11151	*ACTB*	60	Housekeeping (cytoskeleton)
PARVG	64098	*ACTB*	60	Housekeeping (cytoskeleton)
CR2	1380	*TP53*	7157	DNA repair/apoptosis/cell cycle regulation
ESR1	2099	*TP53*	7157	DNA repair/apoptosis/cell cycle regulation

[a]This gene is 5-FU metabolism-related.
